# Primary cilium in kidney development, function and disease

**DOI:** 10.3389/fendo.2022.952055

**Published:** 2022-08-22

**Authors:** Yunfeng Bai, Cuiting Wei, Ping Li, Xuefeng Sun, Guangyan Cai, Xiangmei Chen, Quan Hong

**Affiliations:** ^1^ Department of Nephrology, First Medical Center of Chinese People's Liberation Army (PLA) General Hospital, Nephrology Institute of the Chinese People's Liberation Army, State Key Laboratory of Kidney Diseases, National Clinical Research Center for Kidney Diseases, Beijing Key Laboratory of Kidney Disease Research, Beijing, China; ^2^ Institute of Chinese Medicine, Guangdong Pharmaceutical University, Guangzhou, China

**Keywords:** primary cilium, kidney development, renal disease, renal function, ciliopathy

## Abstract

The primary cilium is a hair-like, microtubule-based organelle that is covered by the cell membrane and extends from the surface of most vertebrate cells. It detects and translates extracellular signals to direct various cellular signaling pathways to maintain homeostasis. It is mainly distributed in the proximal and distal tubules and collecting ducts in the kidney. Specific signaling transduction proteins localize to primary cilia. Defects in cilia structure and function lead to a class of diseases termed ciliopathies. The proper functioning of primary cilia is essential to kidney organogenesis and the maintenance of epithelial cell differentiation and proliferation. Persistent cilia dysfunction has a role in the early stages and progression of renal diseases, such as cystogenesis and acute tubular necrosis (ATN). In this review, we focus on the central role of cilia in kidney development and illustrate how defects in cilia are associated with renal disease progression.

## Introduction

Cilium emanates from the mother centriole, and it is classified into motile cilia (9 + 2 structure) and nonmotile cilia (9 + 0 structure). Motile cilia have nine peripheral doublets of microtubules with a central pair complex (9 + 2), and primary cilia lack the centrally located pair (9 + 0) (1, 2). In vertebrates, cilia are widely distributed. Motile cilia are distributed on the surface of cells of the cerebral ventricle, respiratory mucosa, and reproductive system, and primary cilia are mainly distributed in embryos, kidneys and retina. They play fundamental roles in the asymmetric development of organs, mucus clearance of the respiratory tract, hearing, neurogenesis, and sperm motility (3–7).

**Figure 1:**
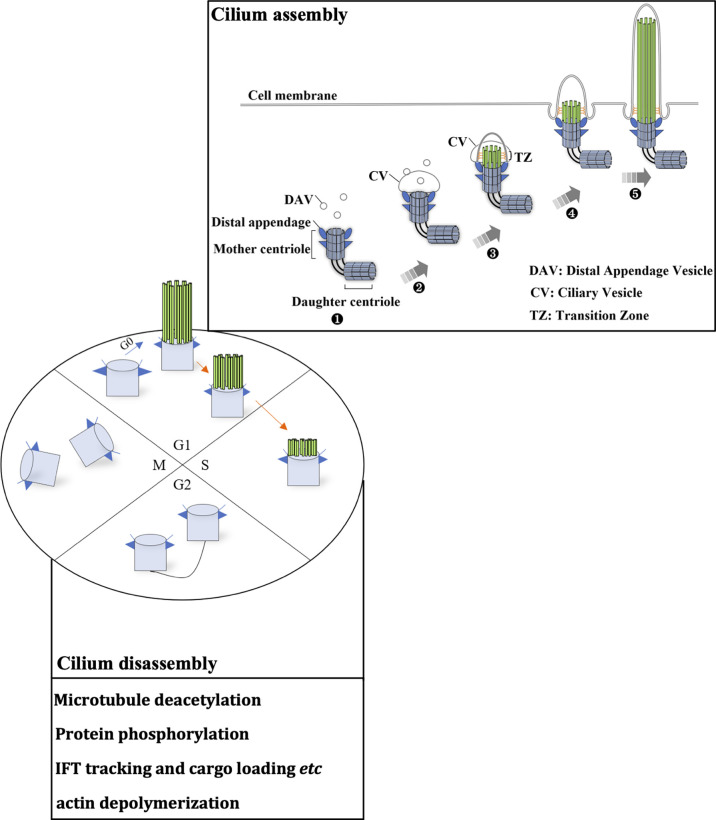
The ciliary life cycle is in tune with the cell cycle. Ciliogenesis occurs in the G0/early G1 phase or differentiation stage. Each stage of the cell cycle is as indicated (G1, S, G2 and M phase), and blue and orange arrows indicate cilium assembly and disassembly, respectively. The mother centriole (light blue cylinder) can initiate ciliogenesis (cilium assembly). (1) Distal appendage vehicles (DAVs, dark blue triangles) accumulate near the distal appendage of the mother centriole. (2) DAVs aggregate and fuse with the mother centriole, forming a ciliary vehicle. (3) Assembly of the transition zone (TZ). (4) The ciliary axoneme (indicated by parallel green rods) covered by CV elongates vertically and fuses with the cell membrane. (5) Extension of the ciliary axoneme and membrane. These microtubule structures disassemble as cells progress to S phase. Several key signaling pathways that mediate cilia disassembly are summarized in the box. During the later S phase, centrosomes begin to duplicate. After mitosis, centrosomes set out to assemble primary cilia.

The cilia life cycle is tightly related to the cell cycle (8–11) and consists of cilium assembly and cilia disassembly. The diverse roles of the primary cilium depend on the well-established balance between cilia assembly and disassembly (**Figure 1**). Cilia assembly is a precise and orderly multistep process. In the absence of mitogen or stimulation by differentiation signals, cells escape from the mitotic phase and enter the G0 phase. Then, cilia begin to assemble. First, within a few minutes of mitogen deprivation, vehicles originating from the Golgi or recycling endosomes, distal appendage vesicle (DAV), cluster at the distal appendage of the mother centriole (MC). This initiates the conversion from MC to the basal body, building a platform for cilia assembly. DAVs aggregate and fuse at the mother centriole to form the ciliary vesicle (CV) (12). CV formation marks the maturation of the basal body. The distal accessory structure protein Cep164 helps to maintain the integrity of this structure and anchors its fusion with the ciliary vehicle by binding to the GTP enzymes Rab8 and Rabin8 (13, 14). In the second step of cilia assembly, Cep164 recruits TTBK2 to the mother centriole. Proper localization of TTBK2 is required for the disappearance of the key repressor CP110 from the mother centriole, which thereby recruits the intraflagellar transport protein (IFT) complex. This complex is responsible for bidirectional protein cargo transport along axonal microtubules and thus promotes axoneme assembly (13, 15, 16). In this process, kinesin 2 carries the cargo from the tip to the base of the cilium, while dynein functions in cargo transport on the opposite side. After CV maturation, the transition zone (TZ) begins to assemble. It then embeds into and enlarges the CV. Subsequently, the ciliary axoneme is wrapped by CV and extends longitudinally. It is covered by the ciliary sheath, and finally fuses with the cell membrane. Ultimately, the mature ciliary composition includes axonemes composed of microtubules and associated proteins, the ciliary membrane connected to the cell membrane, and various matrices between the axonemes and the membrane (8).

Compared to cilium assembly, the signaling pathways for cilia disassembly are poorly understood (17). The Aurora A-HDAC6 and Nek2-Kif24 pathways and actin polymerization are the key signaling pathways that can induce cilia disassembly. It is widely accepted that Aurora A is the major pathway for the direct induction of ciliary microtubule deacetylation through the activation of HDAC6 (18). Nek2 ensures Kif24 activation in cells that lack cilia, and Nek2 is mainly expressed during the S and G2 phases (19). Imbalance between cilium assembly and disassembly leads to the loss of cell cycle regulation, and the loss of cilia may be an initiating factor of the oncogenesis of renal cancer, melanoma, and breast, pancreatic and prostate cancer (20–22).

## Cilia in kidney development and function

Ciliary membranes are rich in receptors and ion channels that can be activated by mechanical or chemical stimuli (23). The proper spatiotemporal localization of receptors and the coordinated transportation of related signal modules that localize to the cilium lay the foundation for cilia sensory function (1, 24). The cilium is an important nexus for Hedgehog signaling, Wnt signaling, GPCR signaling and transforming growth factor-β (TGF-β)/bone morphogenetic protein (BMP) signaling (1, 25, 26). In nephrogenesis, Wnt signaling is of great importance (26). The Wnt signaling branches to β-catenin-dependent (canonical) and β-catenin-independent (noncanonical) pathway.

**Figure 2:**
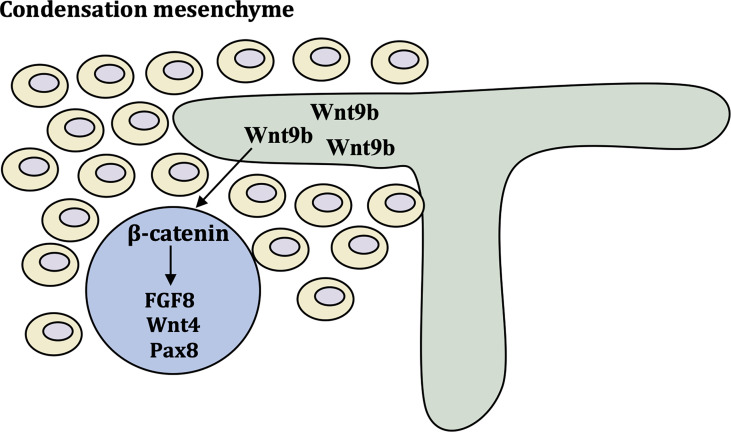
Molecular control of early kidney development. Wnt9b is expressed in ureteric bud and turns on differentiation markers FGF8, Wnt4 and Pax8 *via* β-catenin pathway. These cells form the pre-tubular aggregate highlighted by the expression of critical differentiation factors Pax8 FGF8 and Wnt4.

Wnt9b is expressed in the stalk of the ureteric bud (UB) as it invades and branches into the metanephric mesenchyme (MM) and acts as a paracrine signal to induce the expression of tubulogenic pathway markers, such as fibroblast growth factor-8 (FGF8), Wnt4 and Pax8 (27).. Wnt9b is required for the planar cell polarity (PCP) signaling pathway (28). Wnt4 was detected in condensing mesenchyme, pre-tubular aggregates (**Figure 2**) (29). Inactivation of FGF8 block formation of Wnt4-expressing pre-tubular aggregates, which led to S-shaped bodies, the precursor of nephron cannot develop (30, 31). Wnt9b and Wnt4 primarily employ the canonical, β-catenin-dependent pathway. Studies have shown that β-catenin activation is necessary and sufficient to initiate the tubulogenic program and induce MM in *Wnt9b^-/-^* and *Wnt4^-/-^* mice. However, it is important to maintain a proper balance of canonical Wnt signaling activity, and constitutive β-catenin activation results in cyst formation in all tubular segments (32). *Ksp-cre* conditional inactivation of *APC*, which enhances β-catenin activity, results in cystic kidneys in all tubular segments and a hyperproliferative epithelium (33). Module component jouberin (JBN)-deficient mice show cystic kidneys and malformations of the central nervous system caused by dysregulated Wnt-β-catenin signaling (34).

**Figure 3:**
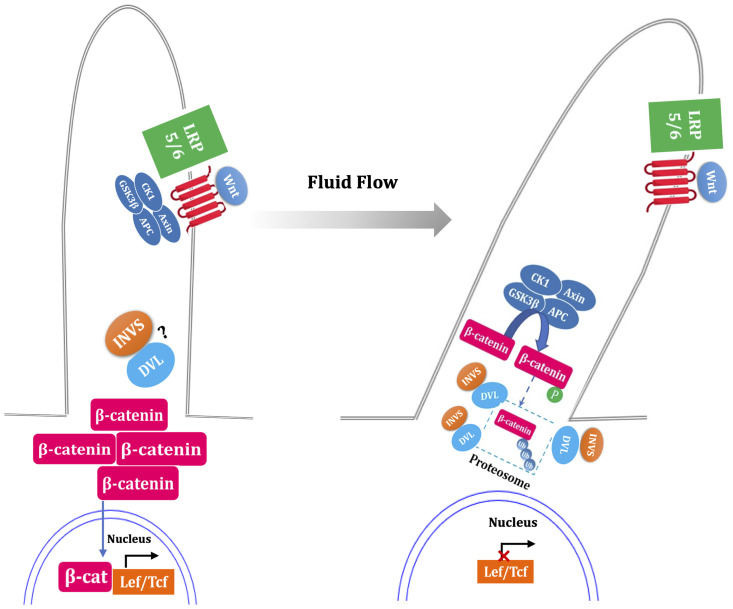
Fluid flow flip the switch from canonical to noncanonical Wnt signaling pathway. In the absence of flow, ligand binding with the Frizzled and LRP5/6 complex results in the inactivation of the β-catenin destruction complex (GSK3β, CK1, APC, Axin), led to stabilization of cytoplasmic β-catenin, which translocate to nucleus and coactivate the transcription level of LEF-TCF family. Fluid flow stimulated primary cilia, which is thought to increase the expression of inversin (INVS) and then reduce the cytoplasmic levels of dishevelled (DVL) by increasing its proteasomal degradation pathway. This process is deemed as switch off canonical signal pathway due to the activation of β-catenin destruction complex. The question mark indicate uncertainty as to the INVS-DVL complexes localization. The dotted box mark indicate that whether β-catenin is phosphorylated and ubiquitylated within the cilium is unknown.

Simon et al. suggested that Wnt functions primarily *via* β-catenin-dependent pathways in the absence of flow (35). Flow sensing by the primary cilium is thought to function as a switch from canonical pathway to noncanonical pathway (**Figure 3**). Studies have suggested that the primary cilium inhibit the activity of canonical Wnt signaling (perhaps promoting noncanonical signaling pathway) in mouse embryos, primary fibroblasts and embryonic stem cells (36).

Activation of GPCR signaling, functionally coupled to calcium channels, led to an increase in calcium concentration in the cilia, and cilium was lengthened by mediating actin depolymerization (1). TGF-β signaling induces the shortening of primary cilia in mouse renal tubular epithelial cells (RTECs) and is related to epithelial and mesenchymal transition (37).

In addition, shear stress stimulates lipophagy and mitochondrial biogenesis in RTECs to produce fatty acids that provide substrates for mitochondrial β-oxidation to generate ATP. This ensures an energy supply for the reabsorption of glucose in RTECs, and this process is dependent on the primary cilium (38). Unilateral ureteral obstruction (UUO) in mice reduced fluid flow, and the authors found defects in lipophagy. These defects resulted in lipid droplet accumulation in kidney cortical cells, intensifying the central role of the primary cilium in sensing mechanical stress to regulate mitochondrial activity and lipophagy (38). Miceli and Roccio et al. hypothesized that the primary cilium–autophagy axis plays a key role in the response to shear stress induced by fluid flow (39–41).

Mutations leading to ciliary structure and function defects give rise to multiple organ-involved disorders termed ciliopathies (42–44). These ciliopathies are accompanied by the following phenotypes: retinal degradation, hearing loss, malformation of the central nervous system, and polycystic kidney (45, 46). The significance of the renal cilium is reinforced by the fact that defects in this organelle lead to polycystic kidney disease, Meckel-Gruber syndrome (MKS), Bardet–Biedl syndrome (BBS), nephronophthisis (NPHP) and renal cell carcinoma (RCC) (5, 45–48) (**Table 1**).

**Table 1:** Cilia phenotype in kidney disease.

Kidney disease		Cilia phenotype	Related gene
Acute Kidney Injury		Increase in cilium length at the early stage	—
Cystic Kidney Disease	Polycystic kidney disease(PKD): ADPKD and ARPKD	Absence of cilium in most cases	*Pkd1,Pkd2, PKHD1,Kif3a etc.*
	Cystic Diseases with Interstitial Nephritis: (NPHP, BBS, MKS, Alström syndrome *etc.*)		*NPHP- BBS- MKS-related gene*
Primary Glomerular Disease	IgA nephropathy	—	—
	Membranous Nephropathy	—	—
	Focal segmental Glomerulosclerosis (FSGS)	(1) *TTC21B^-/-^* lead to cilia defect (2) Increase in cilium length compared to healthy control	*TTC21B*
Secondary Kidney Disease	Lupus Nephritis	Morphological alterations (from 9+2 to atypical 8+2 pattern)	—
	Diabetic Kidney Disease	—	—

—: not reported.

## Cystic kidney disease

Cystic kidney diseases are classified into two broad groups (49): (1) polycystic kidney disease (PKD), which includes autosomal dominant polycystic kidney disease (ADPKD) and autosomal recessive polycystic kidney disease (ARPKD), is characterized by large, polycystic kidneys; (2) the group of hereditary cystic diseases with interstitial nephritis that are characterized by small- to normal-size kidneys with tubular atrophy and interstitial fibrosis, including nephronophthisis (NPHP), Bardet-Biedl syndrome (BBS), and Meckel-Gruber syndrome (MKS). The cysts are lined by epithelial cells and filled with fluid and amorphous material.

### Polycystic kidney disease

ADPKD is a genetic disease with a prevalence of 1:1000; it is caused by mutations in *PKD1* and *PKD2 (* 50). *PKD1* and *PKD2* encode polycystin-1 (PC1) and polycystin-2 (PC2), respectively. Colocalization of GPCR PC1 and ion channel PC2 mediates flow-sensitive mechanotransduction in primary cilia and responds to flow by increasing calcium influx (51). Loss of cilia causes PC1 and PC2 to fail to localize to cilia to perform their functions. This results in excessive proliferation and enlargement of kidney epithelial cells, leading to polycystic kidneys. Inactivation of *Pkd1* and other ciliary proteins in adult animals can cause cystic disease. This suggests that cilia are required not only for proper kidney development but also for maintenance of normal function and morphology (52).

ARPKD occurs mainly in infants and young children, with a prevalence of 1:20,000 (53). It is caused by mutations in *PKHD1* and is characterized by cystic dilatations predominantly of the collecting duct. *PKHD1* encodes polyductin/fibrocystin (PD).

### Cystic diseases with interstitial nephritis

Nephronophthisis (NPHP) is an autosomal recessive disease that accounts for 10%-20% of cases of renal failure in children. It is characterized by cystic kidney tubules and interstitial fibrosis with inflammatory infiltrate. NPHP-related genes (*NPHP1, NPHP2/inversin, NPHP3, NPHP4, NPHP5, NPHP6/CEP290, NPHP7/GLS2, NPHP8/RPGRIP1L, NPHP9/NEK8*) have been implicated in NPHP. Unidentified mutated genes still need to be explored in 70% of cases (54).

BBS is a rare autosomal recessive syndrome characterized by postaxial polydactyly, retinitis pigmentosa, intellectual disability, obesity, hypogonadism in men, and a variety of renal abnormalities that include cysts, calyceal clubbing and blunting, tubulointerstitial nephropathy, and dysplastic kidneys. More than 12 genes have been implicated in BBS.

Alström syndrome is a rare autosomal recessive disease caused by mutations in *ALMS1*. It is mainly characterized by retinitis pigmentosa, hearing loss, insulin resistance, and obesity in children. In adult patients, the presentation of hyalinization of tubules and interstitial fibrosis in kidneys are observed. The ALMS1 protein is located at the base of cilia and centrosomes.

### Primary cilium and cystic kidney disease

The first functional evidence linking primary cilium to cystic disease was derived from *Caenorhabditis elegans* IFT88 and its mouse homologue, polycystic kidney disease gene tg737 mutant mice. *Ift88^Orpk/Orpk^* (Tg737) mutant mice have shorter and blunted primary cilia in collecting ducts (55). Genetic repairment of *Ift88^-/-^* cells may possibly restore ciliary length and normalize ciliary function. The *Kif3A* conditional knockout mouse model also suggests that cystic disease can result from disrupting ciliary function and increasing canonical Wnt-β-catenin activity (36, 56). Evidence linking ciliary function to kidney cyst formation and PCP signaling was demonstrated using *Ift20 Hoxb7Cre* conditional mice (57). Wnt signaling activation and a misoriented axis of cell division account for cyst formation. *Ksp-Cre; Wnt9b* mutant mice have few cysts at P1 but many at P10. This is mainly because of a misoriented and random mitotic axis along the tubule in mutants compared to controls, suggesting that the *Wnt9b* mutant links noncanonical Wnt signaling to cyst formation (28). In summary, these findings support that defective PCP signaling plays a key role in cyst formation during kidney development. However, how these processes deregulate cellular orientation to prompt cystogenesis remains elusive.

An overwhelming abundance of data linking to cilia to cystic kidney disease but the causal relationships between them still need to be defined. A possible explanation for the primary cilia anomalies that cause cyst formation is that ciliogenesis is a multistep process. In this process, the NPHP complex, BBSome and over 20 cystoproteins are localized to the centrosome or the base body of cilium (58, 59), and any gene mutation that causes a loss/gain of function leads to defective ciliogenesis. It disables the function of the canonical Wnt-β-catenin or noncanonical Wnt-PCP signaling pathway, or certain proteins fail to localize to the cilium to induce downstream signaling pathways. Such disruptions lead to the overproliferation of epithelial cells and cystogenesis. Recently Hansen et al. elucidated the contribution of ciliary-derived cAMP signalosome to renal cystogenesis, ciliary cAMP signaling activates mTOR signaling and drives cell proliferation, countering the level of cAMP inhibits cyst formation (60). The study unravels a new molecular mechanism promoting PKD and provides new therapeutic targets to the treatment of PKD.

## Acute kidney injury

Acute kidney injury (AKI) is a clinical syndrome of rapid decline in kidney function over a short period of time (a few hours or days), resulting in the retention of metabolic waste products, urea and creatinine (61, 62). Acute tubular necrosis (ATN) represents only one of multiple causes of AKI; it results largely from ischemia–reperfusion injury (IRI) (62). Deficiency of cilia promotes TGF-β-induced EMT and exacerbates the signaling under its pro-fibrotic signals (37), so restoring cilium length or occurrence may be a promising therapeutic target to anti-fibrosis in IRI. Cilia are critical for epithelial repair in renal IRI, indicating a relationship between the change in cilium length and sensitivity in the altered environment of the injured kidney (63, 64).

Elizabeth Verghese et al. identified that acute tubular necrosis causes an increase in the length of renal cilia, modifying their sensory sensitivity during repair (63). Biopsies from human renal transplants suffering ATN showed a dramatic increase in cilium length at 7 days post transplantation and a trend toward the normalization of cilium length at the later stage. A mouse model of ischemia–reperfusion injury (IRI) showed a similar trend. There was an increase in renal cilium length 1 week post-IRI and a return to normalization at 6 weeks. In addition, Jee In Kim et al. reported that the average length of the cilium in the proximal tubule initially shortened after IRI, and the length of cilia increased at 4 and 7 days, facilitating the initiation of the repair mechanism (64). Thus, we summarized the role of the renal cilium in response to injury and repair of damaged tubular epithelial cell reconstruction.

## Primary glomerular disease

Focal segmental glomerulosclerosis (FSGS) is a pathologic diagnostic term that is mainly characterized by sclerosis of part of the glomerular (focal) or part of the glomerular capillary loops (segmental) (65). Evelyne Huynh Cong et al. reported that a homozygous missense mutation in the ciliary gene *TTC21B* causes familial primary FSGS, and knockdown of the *TTC21B* gene product IFT139 in podocytes leads to primary cilia defects and abnormal cell migration (66). Nevertheless, mutations of the ciliary gene TTC21B that lead to primary FSGS indicate a novel cilia function in primary glomerulus disease.

Ivana Solic et al. recently summarized the length of the primary cilium between healthy control and pathologically-changed kidney tissues, including FSGS, CSF and MCDK. In MCDK, CNF and FSGS, cilia were significantly elongated compared to healthy controls (67).

There are still many cilia-related phenotypes and functions that need to be explored in the near future in IgA nephropathy and membranous nephropathy.

## Secondary kidney disease

### Lupus nephritis

Lupus nephritis represents the most severe clinical manifestation of systemic lupus erythematosus (SLE) and leads to a high percentage of morbidity and mortality in patients. The etiology of SLE is characterized by interactions between genetic susceptibility, immune system abnormalities and hormone regulation disorders that result in tolerance disorders and sustained autoantibody production (68). Numerous 9 + 2 cilia and atypical 8 + 2 pattern cilia were found in the kidney biopsy from a patient with lupus nephritis, suggesting that the frequent occurrence of cilia in the lupus kidney results from metabolic and chemical derangements (69).

### Diabetic kidney disease

Diabetic kidney disease (DKD) is one of the most serious complications of diabetes and has become the most common cause of ESRD worldwide (70). Studies have suggested that the pathogenesis of DKD includes an early increase in glomerular ultrafiltrate velocity leading to an increase in mechanical force in the renal tubule lumen, damage to and reduction in podocytes, thickening of the glomerular basement membrane, and expansion of the mesangial membrane (71, 72). Glomerular hyperfiltration is the initial factor contributing to kidney disease in diabetes (71). The high glucose environment also stimulates the activation of the following metabolic pathways (73): (1) the pentose phosphate pathway; (2) the polyol pathway; (3) the hexosamine pathway; (4) the protein kinase C (PKC) pathway; and (5) the advanced glycation end (AGE) pathway. Subsequently, ROS, diglyceride, pyruvaldehyde and lactic acid accumulate and cause cell damage (74). These changes in energy supply and metabolites are collectively termed metabolic reprogramming. A recent study showed that shear stress is transmitted into the cell *via* “antennas” on the surface of RTECs and primary cilia, which direct the metabolic reprogramming of cells to adapt to the environment (38). However, the function of primary cilia in diabetic kidney disease is still unknown.

The primary cilium of RTECs is an important mechanical force sensor for the shear stress, regulates the energy metabolism homeostasis in RTECs to ensure the energy supply for reabsorption function (38). Diabetic kidney disease is characterized by an increase in luminal shear stress induced by a high ultrafiltrate flow rate originating from the glomerulus. we hypothesized that elongated cilium were observed in the RTECs from DKD. It has been shown that glomerular expression of Sirtuin-1 (SIRT1), an NAD+-dependent protein/histone deacetylase, is reduced in human diabetic glomeruli (75), the expression and acetylation of HDAC6 is regulated by Sirt1 (76), so expression of HDAC6 exhibit down-regulated and activity of tubulin deacetylation was inhibited, so we speculated that glomerular hyperfiltration induced the key cilia disassembly regulator HDAC6 down-regulation, promoting cilium elongation and accelerates the progression of diabetic kidney disease. Lipid nanoparticles targeting renal cilium to remote control of cilia movement will be a possible therapeutic target to the diabetic kidney disease.

## Renal cell carcinoma

In patients with RCC with mutations in the VHL (von Hippel–Lindau disease tumor suppressor) gene, primary cilia have been lost, and the re-expression of VHL proteins restored cilia occurrence (77). Ciliogenesis is inhibited in many types of cancer, including renal cell carcinoma (78), prostate cancer (79), pancreatic cancer (80), breast cancer and ovarian cancer (81, 82). Primary cilia are essential for Hedgehog signaling activation during development (1). Abnormal activation of Hedgehog can lead to a variety of tumorigenesis (83–85). The importance of cilium loss in tumorigenesis, maintenance, and progression, as well as chemotherapeutic resistance emerged, which suggesting that restoration of primary cilia in tumor cells may be a potential therapeutic approach.

## Ciliary-targeted therapy technology

Since proper cilium assembly and disassembly are required for embryogenesis and organ function, agents regulating cilia-associated proteins to control cilium length and number may become promising treatments for ciliopathy. The need to develop specific cilia-targeted treatments is urgent. Rajasekharreddy Pala. et al. designed an iron oxide nanoparticle-based and cilia-targeted delivery system to deliver agents specifically to the primary cilia, The hearts of ciliopathic displayed hypertrophy with declined functions in left ventricle because of prolonged hypertension. Magnetic field or fluid flow control cilia and then lead to the increase of Intraciliary and cytosolic Ca^2+^. The CT-Fe_2_O_3_-NPs significantly improved cardiac function in the ciliopathic hypertensive models (86).. Techniques for tissue-specific mRNA delivery and CRISPR-Cas gene editing nanoparticles have been developed (87), so we can specifically rescue the compromised cilia phenotype in ciliopathy by tissue-specific gene editing.

Histone deacetylase 6 (HDAC6), a cytoplasmic enzyme, is the major driver of cilium disassembly, and small molecules that inhibit HDAC6 have been demonstrated to restore the ciliary defective phenotype (88–90). Anti-proliferating agents could also be candidates for polycystic kidney disease due to defective cilia-induced cell overproliferation. In addition, tissue-specific mRNA delivery and the CRISPR-Cas gene editing system could be applied to edit cilia-related genes and may be possible therapeutic targets for ciliopathies.

## Conclusions

In summary, the primary cilium is the center platform that regulates diverse developmental signaling pathways, and its function relies on the control of the precise dynamic balance between cilia assembly and disassembly. More details of these signaling pathways and their involvement in kidney disease remain to be explored. How cilia sense mechanical stimuli is still an ongoing research topic. Ciliary-targeted technology urgently needs to be developed. Insights into ciliary defects in kidney disease will help us identify therapeutic targets for kidney injury relief and provide novel insights into disease mechanisms and ciliopathy intervention.

## Author contributions

YB conceptualized and wrote the manuscript. All authors contributed to the article and approved the submitted version.

## Funding

This work was supported by the National Natural Science Foundation of China (Grant No. 32141005 and 82070741).

## Conflict of interest

The authors declare that the research was conducted in the absence of any commercial or financial relationships that could be construed as a potential conflict of interest.

## Publisher’s note

All claims expressed in this article are solely those of the authors and do not necessarily represent those of their affiliated organizations, or those of the publisher, the editors and the reviewers. Any product that may be evaluated in this article, or claim that may be made by its manufacturer, is not guaranteed or endorsed by the publisher.
